# Acupuncture for systemic therapy-associated insomnia in patients with breast cancer: a systematic review and meta-analysis of randomized controlled trials

**DOI:** 10.3389/fonc.2025.1494929

**Published:** 2025-05-08

**Authors:** Lingling Gao, Ying Sun, Tianyu Luo, Huiying Chen, Shan Huang, Ling Zhu, Meixia Ye

**Affiliations:** Department of Surgery, Shenzhen Hospital (Fu Tian) of Guangzhou University of Chinese Medicine, Shenzhen, China

**Keywords:** acupuncture, insomnia, breast cancer, treatment, meta-analysis

## Abstract

**Background:**

Systemic therapy-associated insomnia is highly prevalent among patients with breast cancer. However, no meta-analysis has explored the efficacy of acupuncture for Systemic therapy-associated insomnia among patients with cancer.

**Methods:**

According to the PRISMA Statement, randomized controlled trials (RCTs) through April 2024 were identified and extracted from PubMed, Embase, and the Cochrane CENTRAL Register of Controlled Trials. The quality of the RCTs was assessed using the Cochrane Systematic Review Handbook 5.1 and its recommended risk-of-bias assessment tool. Two independent investigators screened and extracted the data and performed statistical analysis using RevMan5.3.

**Results:**

Of the total 411 studies identified, 4 RCTs were analyzed. The meta-analysis revealed that acupuncture significantly improved the total sleep time and sleep efficiency relative to wait-list control or sham EA among patients with breast cancer experiencing insomnia after systemic therapy (mean difference [MD] 29.86, 95% confidence interval [CI] 16.20–43.51, P < 0.0001 and MD 4.56, 95% CI 1.84–7.29, P = 0.001), reduced the pittsburgh sleep quality index (PSQI) relative to wait-list control or sham EA with an MD of −0.87 (95% CI −1.60 to -0.15, P = 0.02, I^2^ = 25%) in 4 weeks and an MD of −0.82 (95% CI −1.60 to -0.04, P = 0.04, I^2^ = 12%) in 8 weeks, and reduced the hospital anxiety and depression scale (HADS)-anxiety both in 4 weeks with an MD of −0.85 (95% CI −1.42 to -0.27, P = 0.004, I^2^ = 0%) and in 8 weeks with an MD of −0.94 (95% CI −1.56 to −0.32, P = 0.003, I^2^ = 0%. However, no significant differences in insomnia severity index (MD −2.15, 95% CI −5.07 to 0.78, P = 0.15 and MD −1.48, 95% CI −3.91 to 0.94, P = 0.23), and HADS-depression (MD −0.67, 95% CI −2.32 to 0.99, P = 0.43 and MD −0.63, 95% CI −2.39 to 1.12, P = 0.48) in 4 and 8 weeks were observed between the acupuncture group and the wait-list control or sham EA group.

**Conclusion:**

Acupuncture has a great potential to be used in the management of systemic therapy-associated insomnia in patients with breast cancer. More studies with rigorous designs and larger sample sizes are warranted to verify the efficacy and safety of acupuncture for insomnia among patients with breast cancer.

## 1 Introduction

Breast cancer ranks among the most prevalent malignancies worldwide, requiring systemic therapy (like chemotherapy or endocrine therapy) as the cornerstone of treatment for many patients. However, chemotherapy and endocrine therapy often lead to distressing side effects, particularly insomnia. Physiological, psychological, and adverse reactions of chemotherapy and other factors increase the incidence of sleep disorders by up to 80% in patients with breast cancer during chemotherapy (1), which is significantly higher than that in the general population (2). Untreated insomnia can escalate into several adverse outcomes, including cognitive impairment (3) and diminished quality of life (4).

Despite the recognized significance of addressing insomnia in breast cancer survivors, the efficacy of current therapeutic options remains limited. Although pharmacological interventions are commonly employed (5), concerns regarding their side effects and potential for dependency underscore the need for alternative modalities. The safety and efficacy of acupuncture for managing insomnia in cancer-free populations have already been confirmed (6). D’Alessandro et al. confirmed that acupuncture improved sleep in patients with breast cancer (7). In recent years, related randomized controlled trials (RCTs) have examined the effect of electroacupuncture on insomnia after chemotherapy for breast cancer; however, the results are inconsistent (8, 9).

Therefore, in this study, we aimed to conduct a systematic analysis of existing literature and a meta-analysis to evaluate the efficacy and safety of acupuncture in the treatment of systemic therapy -related insomnia in patients with breast cancer.

## 2 Methods

### 2.1 Protocol and guidance

This study adhered strictly to the Preferred Reporting Items for Systematic Reviews and Meta-Analyses reporting guidelines (10), ensuring consistency and transparency. As this study involved a literature review and meta-analysis, ethical approval or informed consent was waived.

To identify relevant RCTs exploring the effectiveness of acupuncture or electroacupuncture (EA) on insomnia after systemic therapy for breast cancer, a meticulous literature search was conducted. This search encompassed PubMed, Embase, and the Cochrane CENTRAL Register of Controlled Trials from their inception dates up to April 2024. The search strategy employed a combination of keywords: (“Breast cancer,” “Breast Neoplasm,” or “Breast Tumor”), (“electroacupuncture,” “Electro-acupuncture,” “acupuncture,” or “Electro acupuncturing”), and (“Insomnia” or “sleep”). To ensure the inclusivity and comprehensiveness of this study, the reference lists of published systematic reviews and RCTs were also screened to identify any potentially overlooked studies.

### 2.2 Inclusion criteria and study selection

The inclusion criteria were as follows: (1) RCTs exploring the therapeutic effects of acupuncture or EA on insomnia after systemic therapy for breast cancer (type of research), (2) patients diagnosed with breast cancer stages I–IV who are currently undergoing systemic therapy or have completed it (participant population); and (3) the experimental group must receive acupuncture or EA, whereas the control group received sham or in the wait-list (intervention measures).

The exclusion criteria were as follows: (1) studies including patients with other interventions (such as auricular acupuncture, auricular acupressure, or transcutaneous electrical acupoint stimulation), the theoretical basis or operational methods of these intervention measures are different from acupuncture. (2) incomplete original text or the data cannot be extracted, and (3) non-RCTs, systematic reviews, comments, editorials, letters, conference abstracts, and animal trials.

### 2.3 Data extraction

Data extraction was independently conducted by two reviewers, utilizing a standardized data extraction form to gather pertinent information. Extracted data encompassed details such as the primary author’s name, year of publication, country of origin, study type, baseline characteristics of the participants (including population size, age), procedural specifics of the treatment and control groups, outcome indicators, and other relevant literature data. If discrepancies arose, a third reviewer was consulted to make a final decision and resolve any differences.

### 2.4 Outcomes

To assess the effect of acupuncture or EA on insomnia after systemic therapy for breast cancer, the following key outcome indicators were evaluated: (1) insomnia severity index (ISI), (2) Pittsburgh sleep quality index (PSQI), (3) total sleep time, (4) sleep efficiency, (5) hospital anxiety and depression scale (HADS)-Anxiety, and (6) HADS-Depression.

### 2.5 Quality assessment

Adhering to the Cochrane Systematic Review Handbook 5.1 and its recommended risk-of-bias assessment methodology, the quality of the included studies was thoroughly examined. This evaluation encompassed scrutiny of various factors, including random-sequence generation, allocation concealment, blinding of participants and personnel, blinding of outcome assessment, incomplete outcome data, selective reporting, and other biased. The results of the risk-of-bias assessment were categorized into “low risk,” “high risk,” and “unclear.” This quality evaluation was conducted independently by two researchers, and any conflicts were resolved through mediation by the corresponding author.

### 2.6 Statistical analysis

The meta-analysis was conducted using Cochrane Collaboration Review Manager (RevMan 5.4). Continuous data were expressed as mean difference (MD) with 95% confidence intervals (CIs), whereas dichotomous data were presented using relative risk with 95% CIs. Results from all studies were pooled to assess differences in efficacy between interventions. The statistical heterogeneity between studies was evaluated using the I^2^ statistic, where an I^2^ value of ≤50% indicated no observed heterogeneity and >50% suggested substantial heterogeneity. A random-effects model was employed for meta-analysis if the included studies demonstrated high methodological quality and no significant heterogeneity.

## 3 Results

### 3.1 Search results

**Figure 1:**
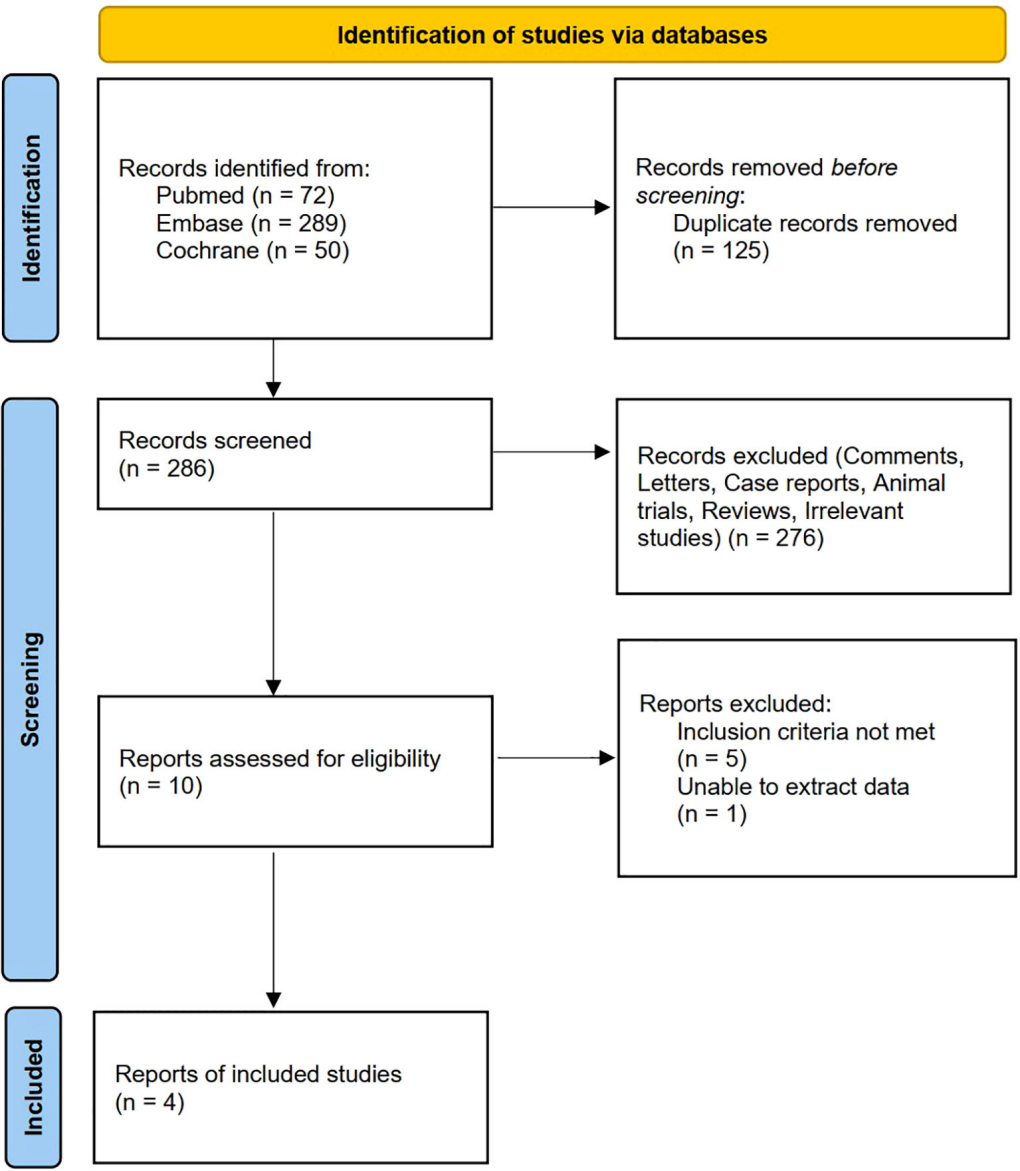
Flow diagram.

Of the initial 411 records retrieved from the electronic databases, 125 were discarded because of duplications. Among the remaining 286 records, meticulous assessment of their titles and abstracts led to the exclusion of 276 ineligible records. Subsequently, a rigorous full-text review of the remaining eight RCTs eliminated another six, narrowing down to four RCTs that fulfilled the eligibility criteria for study inclusion (**Figure 1**). In total, four RCTs (8, 9, 11, 12) involving 278 patients conducted in China and USA, published in 2014 and 2023 were included in the analysis.

### 3.2 Characteristics of studies

The characteristics of the studies included in this meta-analysis are summarized in **Table 1**. The acupuncture group included 15–69 participants, and the sham EA group and wait-list group included 15–69 participants. Not all included studies implemented double-blind designs, and substantial variations existed in acupuncture protocols across trials. These methodological differences contributed to observable heterogeneity among the studies. **Table 1** also provides a comprehensive overview of the acupoints and Intervention regimen used in the included studies.

**Table 1:** Characteristics of included trials.

First Author	Country	Study type	Number of participants^a^	Age/years^a^	Systemic therapy	Groups^a^	Intervention	Acupoints	Intervention regimen	Outcomes
Mao et al. (2014) (11)	USA	RCT	21 vs 20 vs 22	57.5±10.1 vs 60.9±6.5 vs 60.6±8.2	endocrine therapy	EA vs Sham-EA vs Wait-list	EA	SP6, and ST36	2 a week (first 2 weeks), 1 a week (from 3 to 8 weeks); 30 min/session	②⑤⑥
Bao et al. (2014) (12)	USA	RCT	23 vs 24	61 vs 61	endocrine therapy	A vs Sham-A	A	CV4, CV6, CV12, LI4, MH6, GB34, ST36, KI3 and BL65	8 a week (8 weeks); 20 min/ session	②
Zhang et al. (2021) (8)	China	RCT	15 vs 15	52.5±8.9 vs 52.7±6.3	chemotherapy	EA vs Wait-list	EA	EX-HN1, GV20, GV24, PC6, KI3, and SP6	2 a week (6 weeks); 25 min/ session	①②③④⑤⑥
Zhang et al. (2023) (9)	China	RCT	69 vs 69	51.7±9.6 vs 52.7±8.3	chemotherapy	EA vs Sham-EA	EA	EX-HN1, GV20, GV24, PC6, KI3, and SP6	2 a week (first 6 weeks), 1 four weeks (from 7 to 18 weeks); 25 min/session	①②③④⑤⑥

a Present as intervention versus control groups

Outcome: ① Insomnia Severity Index (ISI), ② Pittsburgh Sleep Quality Index (PSQI), ③ total sleep time(TST) recorded by sleep diary, ④ sleep efficiency (SE) recorded by sleep diary, ⑤ Hospital Anxiety and Depression Scale (HADS)- Anxiety, ⑥ Hospital Anxiety and Depression Scale (HADS)- Depression.

RCT, randomized controlled trial; EA, Electroacupuncture; A, Acupuncture; SP6, Sanyinjiao; ST36, ZusanLi; CV4, Guanyuan; CV6, Qihai; CV12, Zhongwan; LI4, Hegu; MH6, Yinweixue; GB34,

Yanglingquan; KI3 Taixi; BL65, Shugu; EX-HN1, Sishencong; GV20, Baihui; GV24, Shenting; PC6, Neiguan.

### 3.3 Risk-of-bias assessment

**Figure 2:**
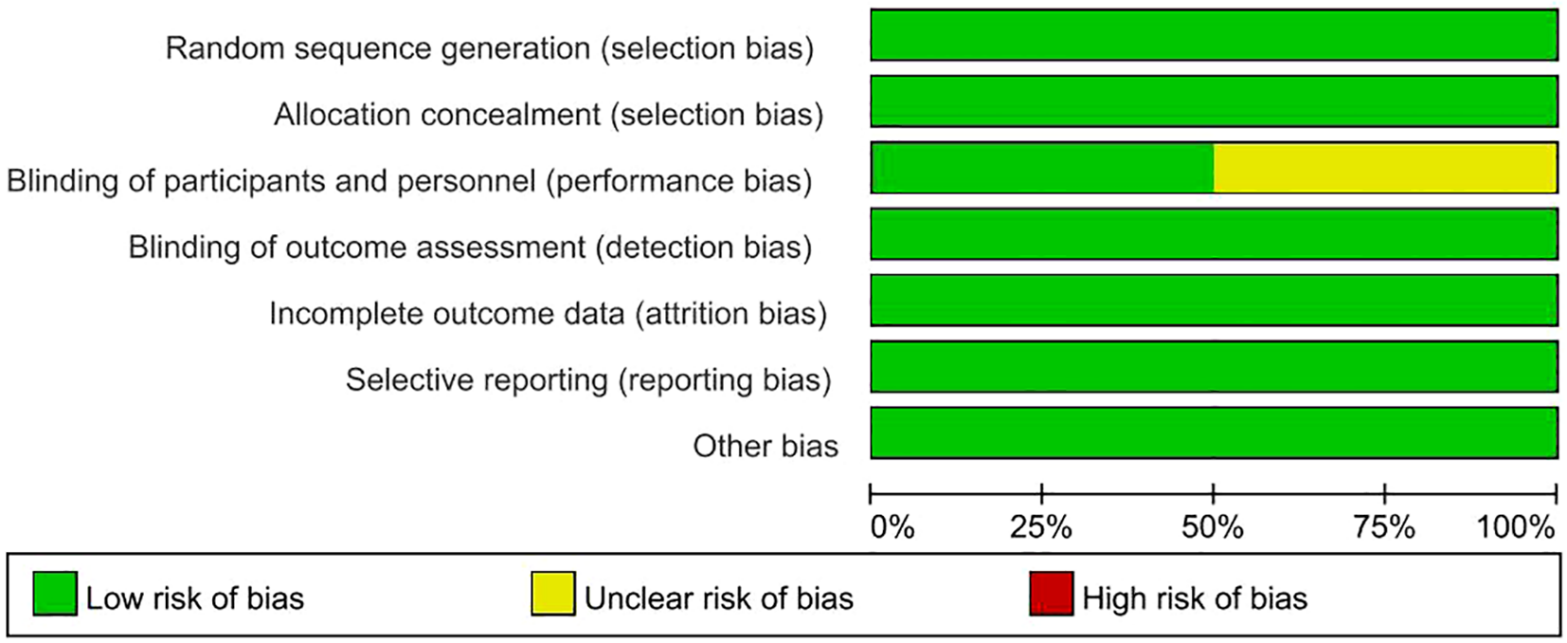
Risk of bias graph.

**Figure 3:**
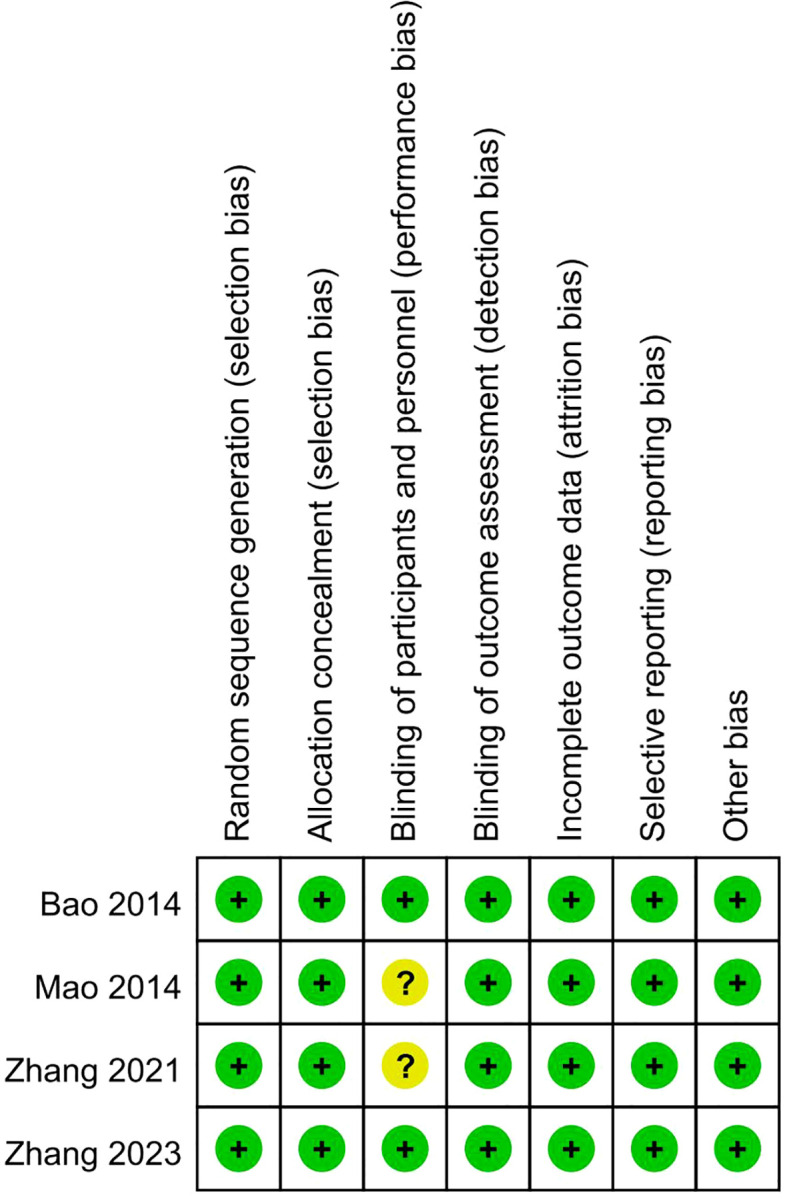
Risk of bias summary.

After conducting a quality assessment using the Cochrane Risk-of-Bias Assessment Tool on the 4 included publications, the overall quality of the entire literature was good (**Figures 2**, **3**).

### 3.4 Outcomes

The analysis encompassed data from 278 participants, divided into the acupuncture or EA group and the sham EA or wait-list group.

### 3.5 Insomnia severity index

**Figure 4:**
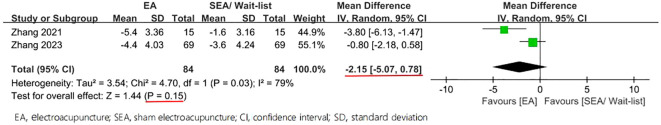
Forest plot of comparison on ISI (week-3).

**Figure 5:**
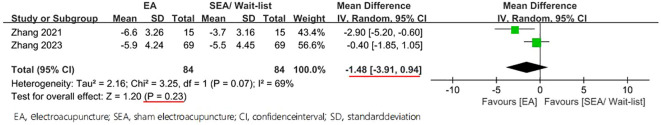
Forest plot of comparison on ISI (week-6).

A meticulous meta-analysis revealed no statistically significant reduction in the insomnia severity index in the EA group compared with the sham EA or wait-list group, neither at 3 weeks nor at 6 weeks with MDs of −2.15 (95% CI −5.07 to 0.78, P = 0.15, I^2^ = 79%) and −1.48 (95% CI −3.91 to 0.94, P= 0.23, I^2^ = 69%), respectively (**Figures 4**, **5**).

### 3.6 Pittsburgh sleep quality index

**Figure 6:**
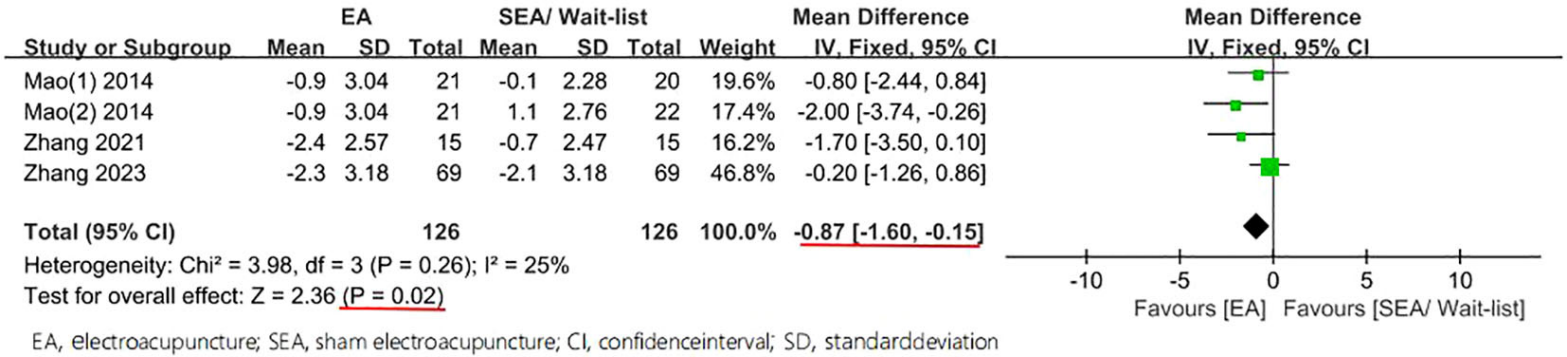
Forest plot of comparison on PSQI (week ≤ 4).

**Figure 7:**
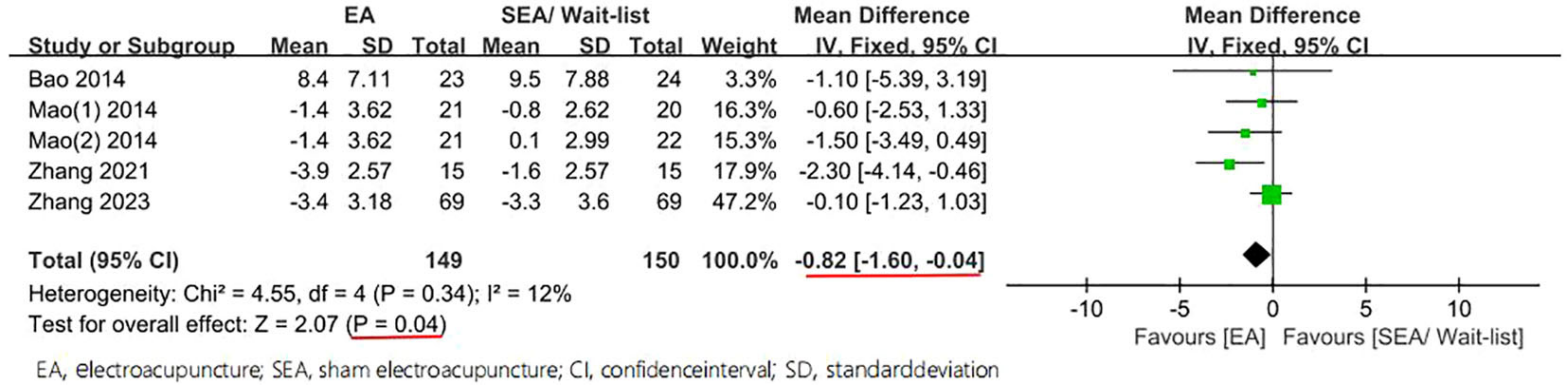
Forest plot of comparison on PSQI (week ≤ 8).

Moreover, statistically significant reduction in the PSQI was found among the groups, with an MD of −0.87 (95% CI −1.60 to -0.15, P = 0.02, I^2^ = 25%) in 4 weeks and an MD of −0.82 (95% CI −1.60 to -0.04, P = 0.04, I^2^ = 12%) in 8 weeks (**Figures 6**, **7**).

### 3.7 Total sleep time, sleep efficiency, HADS-anxiety and HADS-depression

**Table 2** summarizes the results of the analyses of the total sleep time, sleep efficiency, HADS-anxiety, and HADS-depression. This meta-analysis revealed a statistically significant increase in the total sleep time and sleep efficiency among the groups in 8 weeks, with MDs of 29.86 (95% CI 16.20–43.51, P < 0.0001, I^2^ = 0%) and 4.56 (95% CI 1.84–7.29, P = 0.001, I^2^ = 0%). Moreover, statistically significant reduction in HADS-anxiety was found among the groups in 4 weeks, with an MD of −0.85 (95% CI −1.42 to -0.27, P = 0.004, I^2^ = 0%), and a statistically significant reduction was noted in 8 weeks, with an MD of −0.94 (95% CI −1.56 to −0.32, P = 0.003, I^2^ = 0%). Both in 4 and 8 weeks, this meta-analysis revealed no statistically significant reduction in HADS-depression among the groups, with MDs of −0.69 (95% CI −1.65 to 0.28, P = 0.16, I^2^ = 54%) and −0.93 (95% CI −2.12 to 0.27, P = 0.13, I^2^ = 64%).

**Table 2:** Findings of the total sleep time, sleep efficiency, HADS- anxiety and HADS- depression.

Outcomes	Data sets	Participants(n)	Effect estimate; MD [95% CI]	P	I ^2^
Week≤4					
HADS- Anxiety	4	126 vs 126	-0.85 (-1.42 to -0.27)	P=0.004	0%
HADS- Depression	4	126 vs 126	-0.69 (-1.65 to 0.28)	P=0.16	54%
Week≤8					
TST	2	84 vs 84	29.86 (16.20 to 43.51)	P<0.0001	0%
SE	2	84 vs 84	4.56 (1.84 to 7.29)	P=0.001	0%
HADS- Anxiety	4	126 vs 126	-0.94 (-1.56 to -0.32)	P=0.003	0%
HADS- Depression	4	126 vs 126	-0.93 (-2.12 to 0.27)	P=0.13	64%

MD, mean difference; CI, confidence interval; HADS, hospital anxiety and depression scale; TST, total sleep time; SE, sleep efficiency

Insomnia Severity Index.

### 3.8 Publication bias and sensitivity analysis

**Figure 8:**
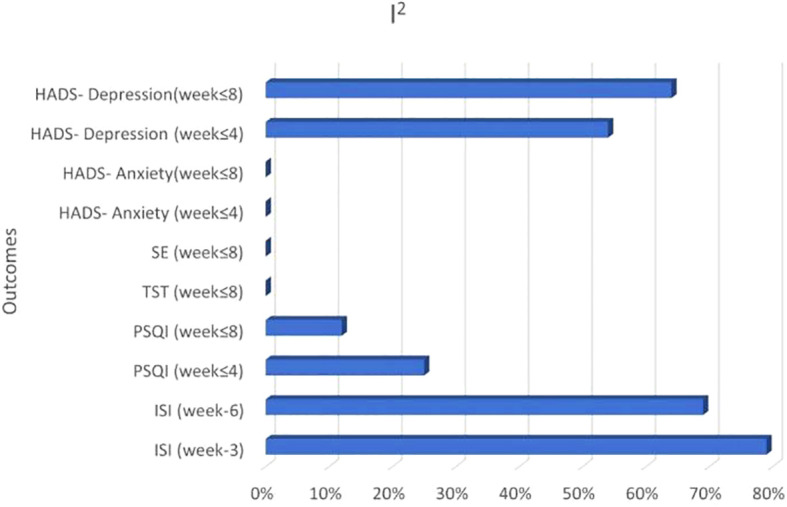
Visualizing the heterogeneity of all outcomes.

Consistent with the guidelines stipulated in the Cochrane Handbook for Systematic Reviews of Interventions, the analysis of publication bias was deemed unnecessary because none of the study groups comprised more than 10 studies. Visualizing the heterogeneity (I ²) of all outcomes reveals that the I ² of the primary outcome measure ISI was higher than that of other outcomes (**Figure 8**). A sensitivity analysis was conducted to evaluate the stability of the findings by systematically excluding individual studies one at a time from the meta-analysis. However, sensitivity analysis could not be performed for the ISI outcomes in either the Week 3 or Week 6 groups due to the inclusion of only two studies in these analyses. The analysis demonstrated variability in the pooled effect estimates for PSQI outcomes across both the group of ≤4 weeks and group of ≤8 weeks, with the studies by Mao (2014) and Zhang (2021) identified as primary contributors to this heterogeneity. A comprehensive comparative examination is warranted to clarify methodological or clinical discrepancies between these two studies and the remaining included studies, as well as to elucidate the underlying factors driving their disproportionate influence on the results. Detailed outcomes of the sensitivity analyses are summarized in **Tables 3** and **4**.

**Table 3:** Sensitivity analysis results after removing one study at a time (PSQI≤4).

Removed study	MD	95% CI	P	I^2^
Mao 2014 (11)	-0.89	-1.70, -0.08	0.03	50%
Mao 2014 (11)	-0.64	-1.44, 0.16	0.12	1%
Zhang 2021 (8)	-0.71	-1.51, 0.08	0.08	34%
Zhang 2023 (9)	-1.47	-2.46, -0.47	0.004	0%

MD, mean difference; CI, confidence interval.

**Table 4:** Sensitivity analysis results after removing one study at a time(PSQI ≤ 8).

Removed study	MD	95% CI	P	I^2^
Bao 2014 (12)	-0.81	-1.61, -0.02	0.04	34%
Mao 2014 (11)	-0.87	-1.72, -0.02	0.05	33%
Mao 2014 (11)	-0.70	-1.55, 0.15	0.10	26%
Zhang 2021 (8)	-0.50	-1.36, 0.36	0.25	0%
Zhang 2023 (9)	-1.47	-2.54, -0.40	0.007	0%

MD, mean difference; CI, confidence interval.

## 4 Discussion

The current meta-analysis, encompassing four studies involving a collective sample size of 278 patients, revealed numerous positive outcomes associated with acupuncture or EA employed in patients with insomnia after systemic therapy for breast cancer. Notably, in comparison with the sham EA or wait-list group, the acupuncture or EA group had increased total sleep time and sleep efficiency and decreased PSQI and HADS-anxiety. However, no significant difference in the insomnia severity index and HADS-depression was found. Sensitivity analysis on the outcome of PSQI showed that Mao (2014) and Zhang (2021) had an impact on the results. We further analyzed and found that unlike other studies, Mao (2014) and Zhang (2021) had different grouping methods in their research, were randomly allocated to the Electroacupuncture or wait-list control group. Meanwhile, these two studies suggested that the conclusions regarding PSQI were significance or close to significance. Therefore, the sensitivity analysis results changed when these two studies were excluded. While our analysis included only four studies, all were rigorously designed RCTs featuring either sham-controlled or wait-list-controlled groups. The methodological rigor of these selected studies enhances the robustness and generalizability of our conclusions.

Breast cancer has the highest incidence and mortality rate among women, and chemotherapy is one of the most important components of the comprehensive treatment for breast cancer. Patients with breast cancer often experience sleep disorders during chemotherapy because of various factors, including physiological, psychological, and adverse reactions to chemotherapy. Regarding physiology, studies have reported a higher incidence of sleep disorders among younger patients with breast cancer undergoing chemotherapy because younger patients may carry a heavier psychological burden than older patients (13). From the psychological perspective, the incidence of anxiety and depression is significantly higher among patients with malignant tumors than in healthy individuals, and the likelihood of anxiety and depression is higher among patients with breast cancer than those with other malignant tumors (14). Depression affects the subjective sleep quality of patients with breast cancer undergoing chemotherapy. Shorofi et al. (15) examined sleep quality in patients with breast cancer undergoing chemotherapy using PSQI and found that the total PSQI score and multiple factor scores were significantly positively correlated with the total scores of anxiety and depression, indicating that the higher the levels of anxiety and depression in patients with breast cancer undergoing chemotherapy, the worse their sleep quality. Among chemotherapy drugs, taxanes are commonly used in breast cancer chemotherapy. To prevent allergic reactions to taxanes, dexamethasone is often used for pretreatment; however, long-term use of large doses of dexamethasone can lead to sleep disorders (16). Chemotherapy drugs can affect ovarian function, causing premenopausal women to experience premature menopause, known as chemotherapy-induced amenorrhea, which can easily lead to sleep disorders (17). During adjuvant chemotherapy, postmenopausal women with breast cancer often report symptoms such as hot flashes, vaginal dryness, depression, and sleep disorders, which can lead to a decline in their quality of life (18). A study that investigated the correlation between sleep disorders and fatigue during chemotherapy for patients with breast cancer revealed that sleep disorders and fatigue often occur as a cluster of symptoms, having a high incidence before and during chemotherapy. Thus, the higher the degree of fatigue, the higher the degree of sleep disorders. Short sleep duration and poor sleep quality can exacerbate fatigue, and the two symptoms are mutually causal, leading to a vicious cycle (19). Adjuvant endocrine therapy is recommended for patients with estrogen receptor-positive or progesterone receptor-positive breast cancer. For postmenopausal patients, aromatase inhibitors (AIs) are the preferred treatment. Research indicates that joint pain associated with AIs is prevalent and significantly impacts patients (20, 21). Additionally, the pain experienced by breast cancer patients can be linked to sleep disorders (22). Therefore, it is important to recognize that patients undergoing adjuvant endocrine therapy may face an increased risk of insomnia as a consequence of their treatment.

Currently, the treatment options for sleep disorders among patients with breast cancer undergoing chemotherapy include pharmacological therapy, behavioral and psychosocial interventions, and physical activities. Any medications used may have potential side effects, and some choices may carry the risk of more severe toxicity and dependency (5). Cognitive behavioral therapy is an evidence-based psychological approach that utilizes behavioral interventions to regulate sleep cycles and cognitive interventions to address sleep disturbances. Palesh et al. used brief behavioral therapy for cancer-related insomnia in patients with breast cancer undergoing chemotherapy and found that the intervention improved insomnia and regulated circadian rhythm (23). Yoga enhances physiological functions and mental health, thereby effectively improving sleep disorders. A meta-analysis conducted by Yi et al. on seven RCTs involving 693 patients with breast cancer undergoing chemotherapy revealed that yoga may help alleviate fatigue, depression, and anxiety in the short term and improve sleep disorders (24). Acupuncture, a traditional Chinese medicine, is widely used to address sleep disorders (25, 26) and has been employed to improve sleep issues among patients with breast cancer (11, 27). Acupuncture is generally safe; a prospective study in Japan involving 65,482 acupuncture treatments did not record significant adverse events (28), and two surveys in the UK encompassing a total of 66,000 treatments did not register serious adverse events (29, 30). Several potential mechanisms have been proposed to explain the effect of acupuncture on cancer-related insomnia. First, acupuncture can regulate brain neurotransmitters associated with sleep regulation (31). Second, the anti-inflammatory effects of acupuncture may contribute to its effect on chemotherapy-related insomnia (32). More research to investigate the mechanisms of acupuncture for cancer-related insomnia are warranted.

Furthermore, this study has several limitations. First, only four RCTs were included, both conducted in China and USA, which may have affected the statistical power and the accuracy of the conclusions. Second, the sample size was small, necessitating larger-scale RCTs to draw reasonable conclusions. Third, the control group settings were inconsistent among the studies, with sham EA and wait-list groups, which may confound the outcomes.

In this meta-analysis involving four studies with 278 patients, active acupuncture regimens demonstrated better total sleep time, sleep efficiency, PSQI and HADS-anxiety than sham control and wait-list among women with breast cancer experiencing systemic therapy -related insomnia. However, no significant differences in insomnia severity index and HADS-depression reduction were found. Overall, active acupuncture can be considered an effective option for treating systemic therapy -related insomnia in patients with breast cancer.

## Data Availability

The original contributions presented in the study are included in the article/supplementary material. Further inquiries can be directed to the corresponding author.
